# Phenotype transition from wild mouflon to domestic sheep

**DOI:** 10.1186/s12711-023-00871-6

**Published:** 2024-01-02

**Authors:** Paolo Mereu, Monica Pirastru, Daria Sanna, Giovanni Bassu, Salvatore Naitana, Giovanni Giuseppe Leoni

**Affiliations:** 1 Dipartimento di Scienze Biomediche, Università di Sassari, 07100 Sassari, Italy; 2 Agenzia FoReSTAS, Regione autonoma della Sardegna, 09123 Cagliari, Italy; 3 Dipartimento di Medicina Veterinaria, Università di Sassari, 07100 Sassari, Italy

## Abstract

The domestication of animals started around 12,000 years ago in the Near East region. This “endless process” is characterized by the gradual accumulation of changes that progressively marked the genetic, phenotypic and physiological differences between wild and domesticated species. The main distinctive phenotypic characteristics are not all directly attributable to the human-mediated selection of more productive traits. In the last decades, two main hypotheses have been proposed to clarify the emergence of such a set of phenotypic traits across a variety of domestic species. The first hypothesis relates the phenotype of the domesticated species to an altered thyroid hormone-based signaling, whereas the second one relates it to changes in the neural crest cells induced by selection of animals for tameness. These two hypotheses are not necessarily mutually exclusive since they may have contributed differently to the process over time and space. The adaptation model induced by domestication can be adopted to clarify some aspects (that are still controversial and debated) of the long-term evolutionary process leading from the wild Neolithic mouflon to the current domestic sheep. Indeed, sheep are among the earliest animals to have been domesticated by humans, around 12,000 years ago, and since then, they have represented a crucial resource in human history. The aim of this review is to shed light on the molecular mechanisms and the specific genomic variants that underlie the phenotypic variability between sheep and mouflon. In this regard, we carried out a critical review of the most recent studies on the molecular mechanisms that are most accredited to be responsible for coat color and phenotype, tail size and presence of horns. We also highlight that, in such a complicate context, sheep/mouflon hybrids represent a powerful and innovative model for studying the mechanism by which the phenotypic traits related to the phenotypic responses to domestication are inherited. Knowledge of these mechanisms could have a significant impact on the selection of more productive breeds. In fact, as in a journey back in time of animal domestication, the genetic traits of today’s domestic species are being progressively and deliberately shaped according to human needs, in a direction opposite to that followed during domestication.

## Background

Among the ungulates, the family Bovidae that includes cattle, goats and sheep, shows the highest level of inter-group diversity with 143 species [1]. The genus *Ovis* is one of the more complex mammalian genera, with regard to its evolution and systematics, showing a high inter-species variability in chromosome numbers ranging from 2n = 52 up to 58, and the presence of hybrids with a number of chromosomes within the same range [2, 3]. Cytogenetic evidence has suggested that the ancestral *Ovis* karyotype had 60 chromosomes, which is still maintained in *Capra* [4]. Based on mtDNA data, the evolution of the *Ovis* karyotype is polyphyletic with both the fission of biarmed chromosomes and the fusion of acrocentric chromosomes being involved [2]. The most common chromosome number is 2n = 54 as in domestic sheep, European mouflon and Cyprus mouflon. The evolutionary split between the *Ovis* and *Capra* genera occurred about 5–7 MYA [5], while the early radiation from which, the current variability within the evolutionary clade including domestic sheep and mouflon originated, dates back to 410 KYA [6].

The genus *Ovis* counts several wild species that currently live in the Nearctic and Palaearctic regions and are classified into three evolutionarily different groups: (i) the Pachyceriforms with the snow sheep (*O. nivicola*), the lean sheep (*O. dalli*) and the bighorn sheep (*O. canadensis*); (ii) the Argaliforms with the argali (*O. ammon*); (iii) the Moufloniforms with urial (*O. vignei*), the Armenian mouflon (*O. gmelini gmelini*), the Anatolian mouflon (*O. g. anatolica*), the Estefahan mouflon (*O. g. isphahanica*), the Laristan mouflon (*O. g. laristanica*), the Cypriot mouflon (*O. g. ophion*) and the European mouflon (*O. g. musimon*) [2, 7–9].

**Fig. 1:**
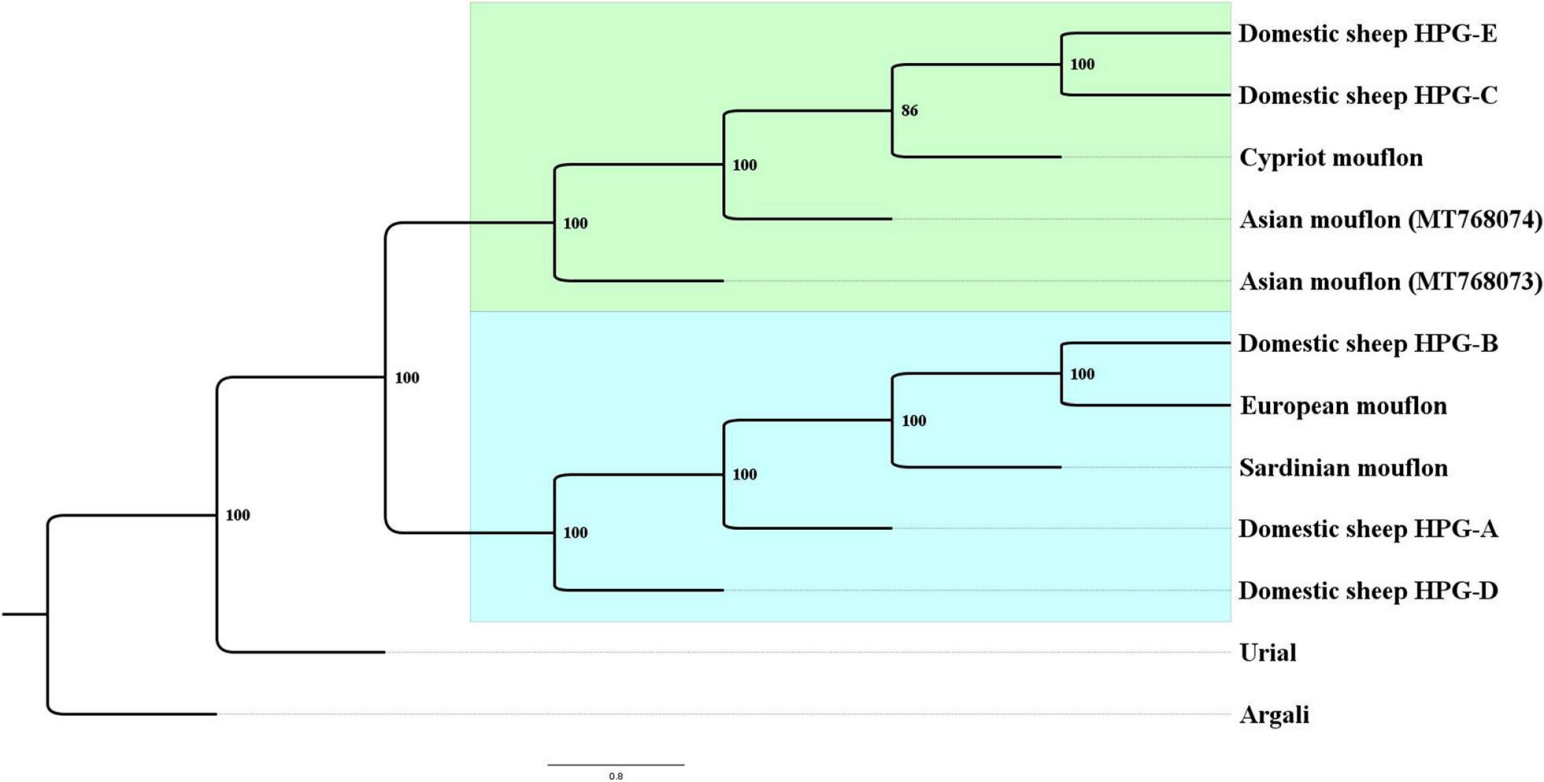
Bayesian tree showing the mitochondrial haplogroups that characterise the current domestic sheep breed variability and the phylogenetic relationships among the wild and domestic species within the sheep/mouflon group. The analysis was performed using the MrBayes 3.2.4 software, assuming 2 million of generations under the T93 + G + I evolutionary model. GREEN: cluster I grouping domestic sheep HPG C and E along with wild mouflons from Near East; BLUE: cluster II grouping domestic sheep HPG A, B and D along with wild European mouflons

Geographical regions where mouflons and urials are both present and can hybridize producing fertile offspring are located in northern [10] and south-eastern Iran, which is consistent with the idea of a single Moufloniform species [9]. More recent studies have disproved this theory by pointing out the existence of two distinct evolutionary lineages for the urial and the group Moufloniforms, the latter being suggested as including the most credited wild ancestor of domestic sheep [6, 11]. The taxonomic classification of the species belonging to the Moufloniforms is a matter of great complexity and for this reason is still under discussion [12]. Genetic studies based on the analysis of small portions of the mitogenome (the control region and/or the cytochrome b gene) and of entire mitogenome sequences of modern domesticated sheep breeds and wild mouflons distributed over a wide geographical range allowed to identify five mitochondrial haplogroups (HPG) named A, B, C, D and E [7, 13–19]. Figure 1 illustrates the phylogenetic tree showing the five HPG that characterise the current domestic sheep breeds and the phylogenetic relationships among the wild and domestic species within the sheep/mouflon group.

More than half of the modern sheep harbour HPG B, followed by HPG A (34%), C (9%), and D and E (< 0.5%). HPG A and B are the most frequent and worldwide spread, with HPG A being particularly frequent in Asian sheep and HPG B in European sheep [14, 19]. In northern and southern Europe, HPG A, B and C were detected with a high preponderance of HPG B (above 90%) and some rare HPG C. The distribution of HPG C is limited to Asia, the Fertile Crescent and Europe [7, 18, 20, 21]. Meadows et al. [17] and Pedrosa et al. [7] proposed that these HPG could be the result of multiple independent domestication events. Indeed, their distribution across different regions might be due to the human-mediated introduction of male individuals from other areas, which then crossed with local breeds. Accordingly, the co-selection of phenotypic or nuclear genetic traits in a specific HPG was probably spread through the high reproductive potential of males. Such a hypothesis is strongly supported by molecular evidence inferred by the combined analysis of mitochondrial DNA (mtDNA) and Y-chromosome markers in domestic sheep and wild mouflon individuals, which suggests that the first breeders ‘upgraded’ local populations by using rams that had different paternal origins and carried the traits to be selected [22]. Another hypothesis is that they could have arisen from a single domestication event that recruited highly divergent wild lineages, as confirmed by a recent study carried out on 57 ancient samples from Neolithic European domestic sheep, where at least three mtDNA haplogroups, A, B and D, were detected, with HPG B being already predominant [23]. Such a result suggests that the rise of the current mitochondrial lineages predates the domestication of sheep, since they were already present at the onset of the domestication process.

Based on the molecular data collected to date, little doubt remains that the mouflon, *O*. *gmelini*, is the maternal origin of domestic sheep [23], with the Asiatic subspecies *O*. *g. gmelini*, *O. g. anatolica* and *O. g. ophion* found to be the most probable ancestors of the sheep carrying HPG C and E, and the Sardinian-Corsican subspecies *O. g. musimon* the most probable ancestor of the HPG B sheep [6, 24, 25].

The Asiatic mouflon, with populations that are currently present in the sub-Caucasian area, from Cyprus and Anatolia to Iran, have been shown to have a large range of genetic intraspecific variability. A recent study based on the analysis of the mtDNA sequences pointed out a close relationship between the European domestic sheep and the Anatolian Neolithic mouflon, the latter showing a higher genetic affinity for the current European domestic HPG than the Anatolian breeds [26].

From the 1700s, the European relative of the Asiatic mouflon, the European mouflon (*O. g. musimon*), was reintroduced in mainland Europe from the Sardinian-Corsican stock [25]. It is considered a remnant of the first moufloniformes that arrived in Corsica and Sardinia ca. 6000 years ago [6, 27].

Once relegated to the rank of feral sheep, the European mouflon has recently regained some of its lost charm thanks to a study carried out on 29 mitogenome sequences including wild and domestic sheep that identified, in the Sardinian mouflon population, the oldest mtDNA haplotype so far described within HPG B [6]. This finding stresses the importance of the Sardinian mouflon population as historical and genetic memory of the wild pool introduced in Sardinia by the first settlers during the Neolithic, which has been probably lost in the mainland Europe populations.

In spite of all this evidence, there is still confusion about the nomenclature of the Mufloniforms species. The main inconsistency comes from the use of the name *O. orientalis* to indicate the progenitor of the modern mouflon and domestic sheep. Indeed, *O. orientalis* was first used to describe the Alborz red sheep, an Asiatic mouflon/urial hybrid population, which is one reason why this name cannot be used and may enter into homonymy [24, 28]. According to the resolutions of the 5th International Symposium on Mouflon held in Cyprus in 2016 [29], and to the IUCN Red List of Threatened Species 2020 [30], the name *Ovis gmelini* is the designated name of the Asiatic mouflon [25], and *Ovis vignei* indicates the urial.

### Domestication: a real evolutionary process?

The archaeological records and the genetic data collected to date suggest that the domestication is an endless process, which probably started 13 KYA ago, first taking place in eastern Anatolia and in northern and central Zagros [31–33]. The epochal change from hunting to farming in ancient civilizations changed humanity forever, leading Neolithic populations to establish permanent settlements. Global warming at the end of the last glacial period and the extinction of large animals such as the *Megacelors* increased the number of mainland European areas available for cultivation and pastoralism [34], and sheep and goats were the first livestock species to be domesticated [35]. The wild sheep living in eastern Anatolia and North-West Iran [36] between 8500 [37] and 12,000 years ago [38] proved ideal for early farmers as a constant and readily available source of meat and skins first, and of milk and wool later [39]. Woolly sheep began to be selected around 6000 BC, becoming quickly predominant and replacing the first sheep [40]. Warmer temperatures and long-term climate stability allowed farmers that travelled from the Middle East towards Europe along the Mediterranean coast or the Danube River, to settle down [35, 41], thus laying the foundations for the beginning of the worldwide diffusion of sheep and other domestic species.

### Transition from wild to domestic phenotype

During the domestication process, a series of morphological, physiological and behavioral traits have been fixed which have led to the current phenotypic differences between wild and domestic species. These traits, including brain and tooth size, ear and tail size and shape, blotchy coloration, hormonal changes and length of the reproductive season, characterize many species that were domesticated in different ways and for different reasons. Moreover, some of these traits have seemingly appeared without deliberate selection. The association between domestication and a set of phenotypic traits was first highlighted by Darwin [42] and has not yet been fully clarified.

In the 1950s, the geneticist Dmitri Belyaev [43] conducted an experiment in Novosibirsk, Russia, that consisted in taming silver foxes (*Vulpes vulpes*) by selectively breeding the friendliest ones, in order to prove, for the first time, that a set of phenotypic traits changes when an animal goes from wild to tame. Although this experiment is controversial and severely criticized by the scientific community, it has provided useful insights for subsequent investigations. In 2014, Wilkins et al. [44] proposed the hypothesis of the “domestication syndrome” (DS) according to which selection for tameness had acted on phenotypic traits that are apparently unrelated to the selected property, across a wide variety of domestic species. Wilkins et al. [45] argued that the traits associated with the DS could be related to changes in formation, differentiation or migration patterns of the neural crest cells (NCC), which in turn could be induced by selection for tameness. Based on this hypothesis, alterations in the genetic regulatory networks that govern the formation and development of NCC could have led to the phenotypic traits which characterize domesticated species compared with their wild relatives [45, 46]. In a previous study, Crockford et al. [47] proposed the “thyroid hormone hypothesis” that relates the domesticated phenotype to an altered thyroid hormone-based signaling. The two hypotheses were later evaluated based on genomic data, and although the NCC hypothesis received much support, they are not necessarily mutually exclusive since they may have contributed differently to the process over time and space [44, 48, 49]. Selection for tameness would therefore have acted indirectly on a broad set of genes and signaling pathways involved in behavior, morphology, and physiology. However, keeping in mind the above hypotheses, it cannot be overlooked that, at least for some traits, human-mediated targeted selection should still be considered as the most plausible hypothesis for the development of a domestic phenotype.

Wilkins et al. [44] listed several candidate NCC-related genes that could be involved in the DS syndrome. In a recent study, a comparative analysis of selection was carried out for most of these candidate genes [50]. The results highlighted signals of positive selection on these key genes in domesticated compared to wild species, which is consistent with the hypothesis of an important role for co-selection of genes in the DS. The main question is: can the domestication process be an example of evolution induced by the adaptive response of species to new human-mediated environmental conditions? In this review, we critically analyse the differences at the level of the main morphological traits that discriminate between domestic and wild sheep, by referring to the most important studies on this topic. The phenotypic traits that are analysed are coat type, tail size and horn shape.

## Main text

### Coat phenotype

#### Wool shedding evidence

Circannual rhythms regulate seasonal reproduction in many vertebrates. In the wild, sheep reproduce in autumn with a gestation length of about five months, so that lambs are born in the spring when the weather is warmer and grass is available. In addition, to cope with the large variations in temperature due to the seasonal cycle, with heat in summer and cold in winter, adaptive mechanisms have been developed to keep the body temperature almost constant. A typical example is represented by the hair follicles, which show a growth phase (anagen) followed by a regression (catagen) and a final shedding phase (telogen) that are functionally linked to seasonal changes. In mouflon, the hair reaches its maximum length in December (≈ 5 cm) followed by a large reduction in summer (≈ 2 cm). Indeed, in line with the seasonal changes in temperature, the mitotic activity increases during the summer-autumn transition and the hair follicles move from the telogen to the anagen phase [51]. The mouflon coat, called “fleece”, is made up of structurally similar brown pigmented hairs, which differ in diameter and length. They are commonly composed of a layer of primary and medullated fibres (P) of variable diameter and length covering a layer of shorter, finer, non-medullated secondary undercoat (S) fibres [51].

**Fig. 2:**
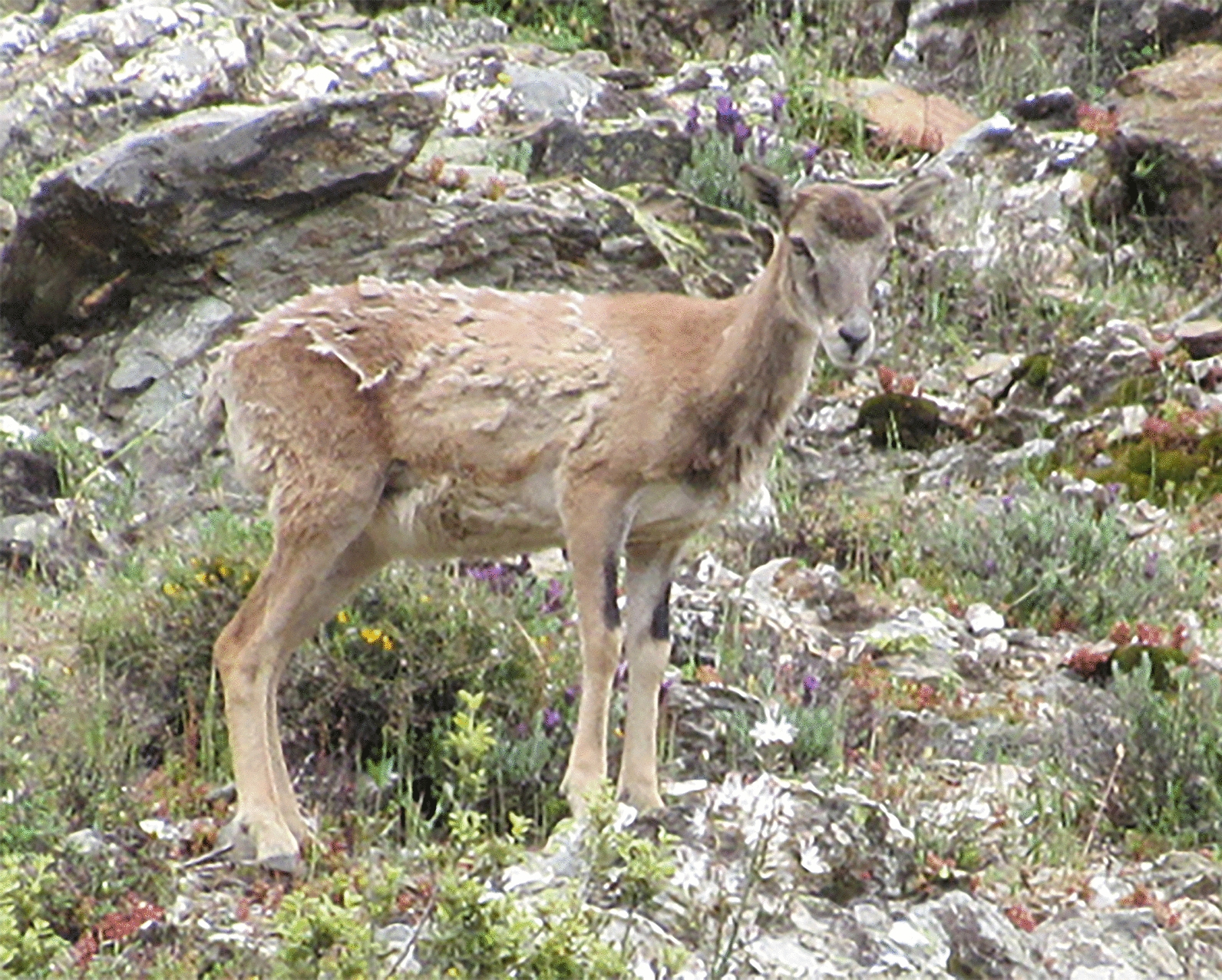
Young female mouflon. The shedding of hair in spring is evident

In the mouflon, the first shedding takes place about five months after birth while the second, very evident, is observed in the following spring (Fig. 2).

It is reasonable to assume that the shedding of wool was the key element in the selection of individuals with continuous fleece growth, which characterises the second wave of sheep domestication, since they are more suited to producing wool for clothing. On the basis of historical reconstructions and genetic analyses that were carried out in recent decades, it is evident that sheep bred for wool, with a single-coated fleece, have gradually replaced the more ancestral sheep with a double-coated fleece [52, 53]. The evolution of the modern sheep from an archaeological perspective [54] occurred on a millennial time scale, with the transition from an animal with a two-layered fleece and an annual shedding to an animal with continuously growing hair. The earliest record of wool production dates back to the 4th millennium BC in the Mesopotamian area, but it is in the Caucasian region of Majkop that the most ancient fragment of archaeological wool-processing fabric was found [55]. Unfortunately, the analysis of fibres and archaeological studies cannot provide detailed information on the quality and characteristics of prehistoric sheep fleece due to the perishable nature of the textile product. In relation to wool production and textile manufacture, one of the most prominent achievements of archaeozoological studies derives from the analysis of slaughtering patterns [40]. It has been demonstrated that differences in the prevailing slaughter age can indicate the primary economic role of sheep at a given site [56, 57]. Indeed, since older sheep normally produce the largest amount of wool [58], their presence in a herd could suggest that the primary aim is wool production. However, the number of published papers on this topic is still limited and further genetic studies on ancient sheep samples are needed.

**Fig. 3:**
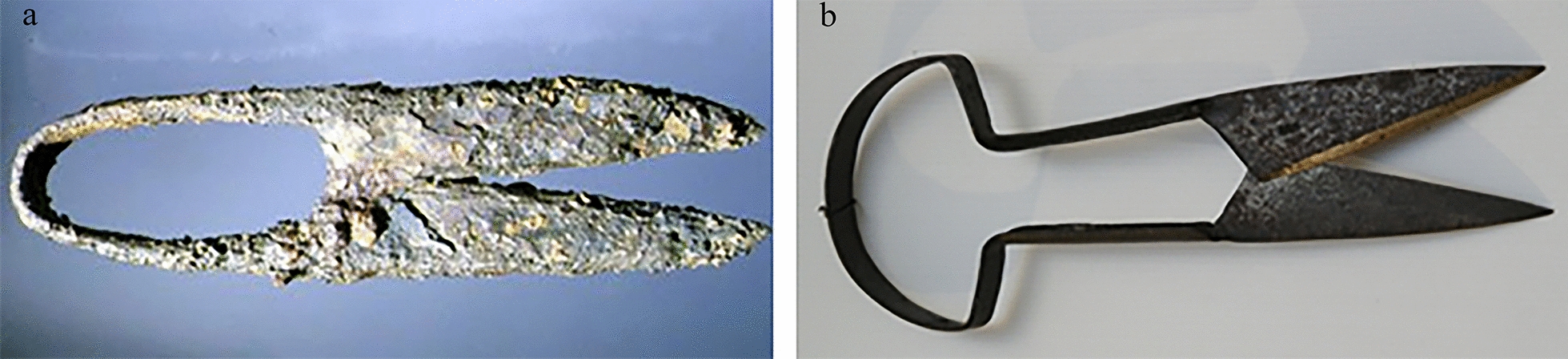
Hand shears for sheep shearing. Find of iron shears used in Roman times (Pompeii museum) (**a**), iron shears currently used (**b**)

As documented by the cuneiform scripts, wool replaced leather as a symbol of royal prestige, and also women played a key role in its production [59]. In the early periods, the wool was worked by hand or with bronze combs, and later, in Roman times, by means of efficient iron shears (Fig. 3).

An analysis and comparison of the P and S fibres have revealed the ability of the follicles to switch from shedding to continuous growth, which is a characteristic of domestic sheep, and even to revert from domesticated to primitive states [60]. The authors of this study hypothesized that the NCC were involved in the morphogenesis of the follicles and the changes associated with the domesticated phenotype, since the timeframes during which the modifications to the coat structure and composition occurred, suggest that they are unlikely to have arisen from random mutations and natural selection. Indeed, NCC influence, among other things, the function of the pituitary, thymus, thyroid and adrenal glands and consequently are responsible for the production of various hormones and neurotransmitters that control several phenotypic and physiological traits [45].

Hair follicle cycling is influenced by a variety of hormones [61, 62], including the pituitary hormone prolactin and thyroid hormones. The concentration of prolactin in plasma could act as a key factor in the modulation of hair length and moult progression [63]. The signaling pathway for the thyroid hormone is also involved in this modulation: disruption of the main thyroid hormone binding isoforms reduces the number of follicles in the anagen phase [64]. Four genes have recently been identified that may be involved in hair changes related to hair follicle formation and wool shedding: *PRX* that regulates the determination of wool properties, *SOX18* that promotes the angio/lymphogenesis and the hair follicle differentiation, *TGM3* that modulates the hair follicle development, and *TCF3* that is expressed in quiescent pluripotential stem cells [65].

#### Coat color

There is a wide range of correlations between coat color and domestication in animals, although the underlying molecular background is not fully clarified. Based on the evidence collected to date, the variation in coat color in domestic animals was probably not a pleiotropic effect of selection for docility, as color varieties probably appeared very soon after the beginning of the domestication process and when humans started to actively select them.

In both preys and predators, the coat color represents an important form of camouflage by homochromia and can be an integral part of social communication and recognition [66]. The standard wild-type sheep coat color is generally dark-bodied with a pale belly, similar to other mammalian wild-type coat color patterns [66]. This wild-type coat color pattern is much rarer in domestic sheep, where coat color is an important breed characteristic and production trait. In domestic breeds, unlike in their wild relatives, the lack of natural selection allows coat color genetic variants to arise and segregate. As a result of artificial selection for white fibres, the white coat phenotype, which is the product of an epistatic autosomal dominant inheritance, has reached a high frequency in certain breeds. Pigment cells in vertebrates have their origin in the neural crest. Melanoblasts, which are a pre-stage of melanocytes, migrate from the neural crest to the epidermis and then into hair follicles. Coat color variation is a complex trait which is probably determined by more than one gene [67]. Coat color is associated with the level of melanin that is synthesized by the melanocytes and then transferred into hairs. Tyrosinase and both the tyrosinase related proteins, TRP-1 and TRP-2, are the rate-limiting enzymes that catalyze melanogenesis and mediate coat color pigmentation [68, 69]. There are two distinct types of melanin: black to brown eumelanin and yellow to reddish-brown pheomelanin. Coat color is determined by the ratio of eumelanin to pheomelanin. At least three genes are involved in the amounts of eumelanin and pheomelanin: *extension (E gene)*, *ASIP* (or *agouti*) and *POMC*, which encode the melanocortin receptor type 1 (MC1R), the agouti signalling protein (ASIP) and the pro-opiomelanocortin, respectively. The latter is the precursor of the alpha-melanocyte-stimulating hormone (α-MSH). ASIP, MC1R and α-MSH act in concert for the production of melanins: when MC1R binds to α-MSH, the level of eumelanin increases leading to a black/brown pigment; when MC1R binds to ASIP, the level of eumelanin is lower, so the relative amount of pheomelanin is higher and a red/yellow pigment is produced [70, 71]. The level of α-MSH is influenced by the seasonal trend with basal concentrations in long photoperiods and, conversely, high concentrations in short photoperiods [72]. ASIP is an endogenous antagonist of α-MSH in several vertebrate species [73]. It is highly conserved in mammals and acts as a competitive inhibitor to prevent α-MSH binding to MC1R, resulting in the inhibition of MC1R signalling and eumelanogenesis [74, 75]. Previous studies documented that the sheep dominant white phenotype is related to a variation in gene copy number and to the presence of polymorphisms in the *ASIP* gene [67]. In some sheep breeds, the white coat color is attributed to a duplication of the *ASIP* gene, as in the case of the Australian Merino sheep. An analysis of the structure of the *ASIP* gene in Merino sheep showed that all white Merinos had at least one duplicated *ASIP* allele, whereas all the recessive black Merinos contained only one allele [76]. It is plausible to assume that the white phenotype was subsequently selected by humans since it was more suitable for dyeing wool.

### A matter of (tail) size: fat vs. thin and long vs. short

The tail phenotype should not be considered to be strictly related to the DS, since there is a large phenotypic diversity of tail patterns observed in domestic species. Phenotypic variations in sheep tails may represent traits that are not directly derived from the neural crest and not necessarily reflecting changes during the initial stage of domestication.

The sheep tail phenotype is a distinctive trait between wild and domestic sheep [77]. The mouflon, which is considered the most credited wild ancestor of sheep, shows a short thin-tail phenotype suggesting that divergent tail phenotypes emerged later. It is believed that the transition from a thin to a fat tail is due to an adaptive response to climate change with increasing temperatures, long periods of soil drought and very low food production [78]. Presumably, ancient breeders selected sheep with fat tails for their adaptability to desert conditions and as a source of fat for cooking. According to this hypothesis, fat-tailed sheep breeds are still preferably raised under local pastoral and arid conditions [79, 80].

Today, the number of fat-tailed sheep has declined significantly. Indeed, the fat tail does not meet the current requests (low fat) of commercial food and farming activities (animal welfare). In addition, a fat tail negatively influences mating [81, 82], locomotion [83, 84] and carcass features [85]. Actually, fat-tailed sheep could deposit up to 20% of their carcass weight as tail fat [85], and for this reason, thin-tailed sheep breeds are increasingly preferred by farmers and butchers because fat tail is considered a waste product [86]. Moreover, breeding fat-tailed sheep is expensive because it requires a surplus of energy, which is turned into fat, and then deposited into the tail. Several studies have been carried out to investigate the underlying molecular mechanism and to identify the specific genomic variants that are responsible for the phenotypic variability of sheep tails. Comparative analyses conducted on fat-tailed and thin-tailed sheep breeds highlighted the *bone morphogenetic protein 2* gene as a potential causative gene for the tail phenotype [87–89]. Another gene probably involved in sheep tail fat deposition is the *platelet-derived growth factor D* (*PDGF-D*) gene [49, 89–95], with the recent investigation of Dong et al. [90] reporting a correlation between mutations that occur within the first intron of the *PDGF-D* gene and the fat tail phenotype. In another study, which includes more than 200 sequenced whole-genomes of wild and domestic sheep, Li et al. [91] found that tail fat deposition is correlated with the level of PDGF-D protein in adipose tissues.

**Fig. 4:**
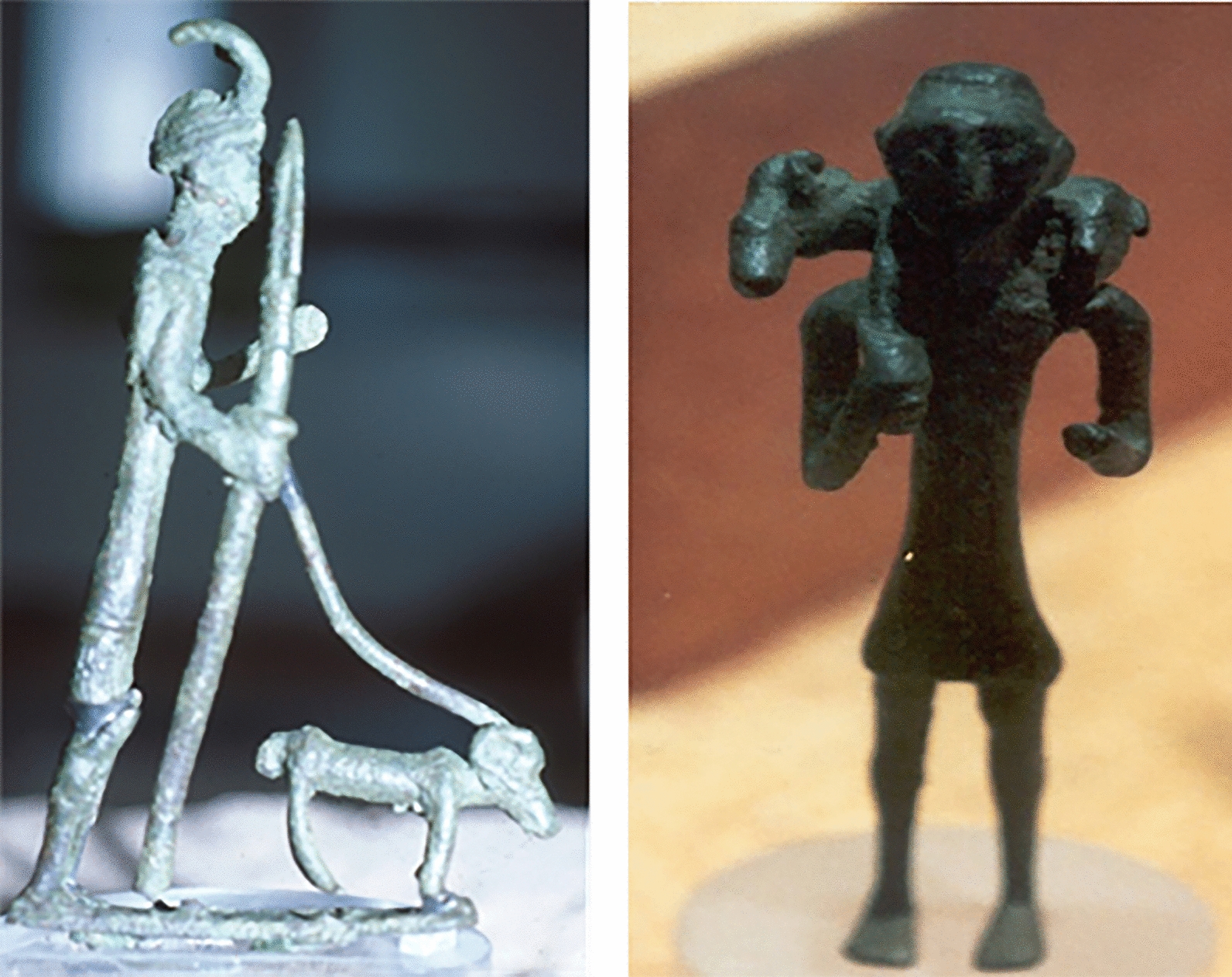
Bronze statues from the Nuragic period (National Archaeological Museum of Cagliari). Pastoral practices are shown. Mouflon is clearly recognizable due to its short tail

**Fig. 5:**
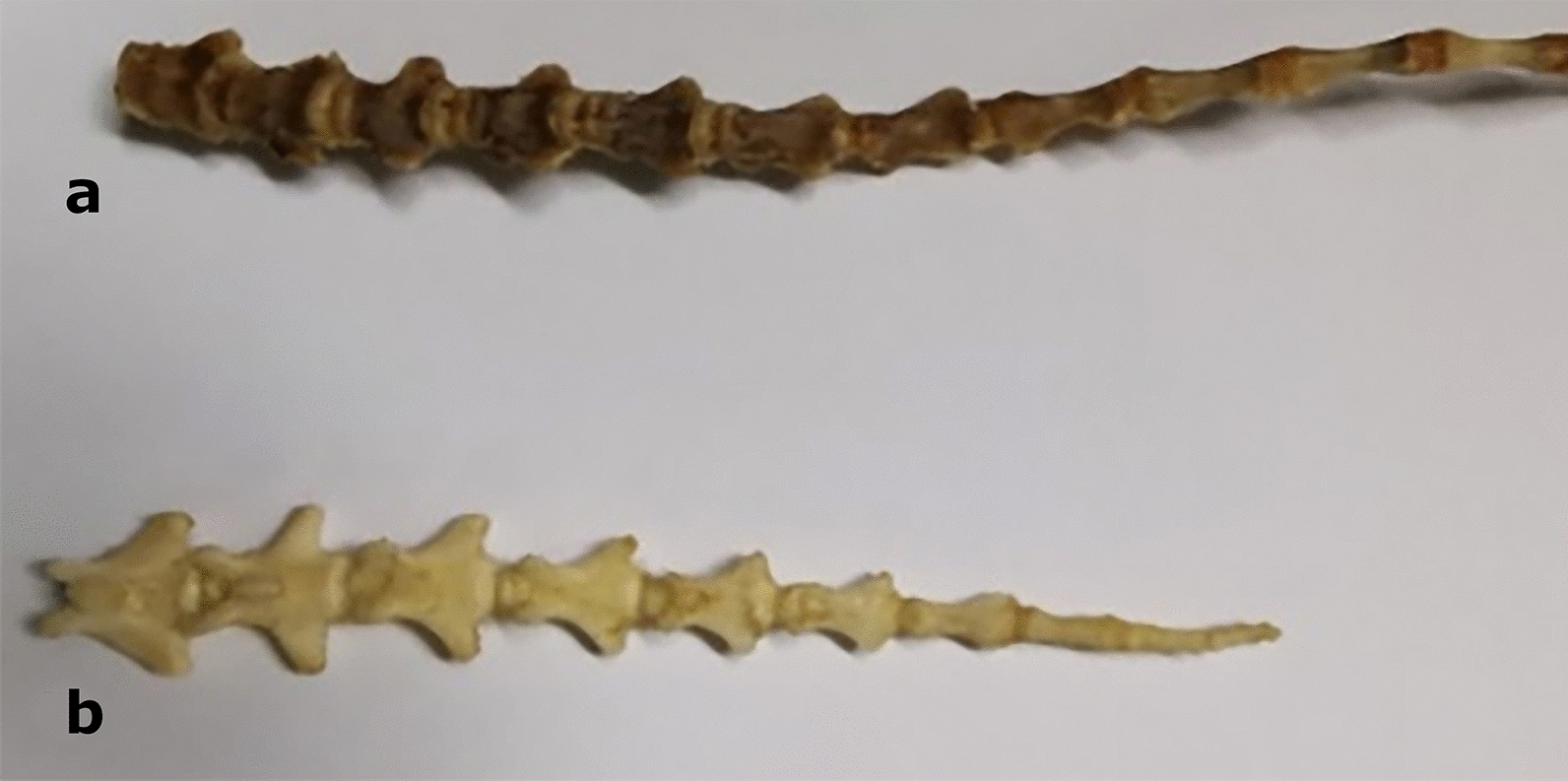
Skeleton bones in the sheep and mouflon’s tail. Sheep’s tail with 22 coccygeal vertebrae (**a**), mouflon tail with 11 coccygeal vertebrae (**b**)

The number of caudal vertebrae determines the length of the tail, which is another phenotypic trait that distinguishes wild and domestic breeds (Fig. 4). The European mouflon has 11 coccygeal vertebrae compared to the 20–24 that are commonly found among the current domestic sheep breeds [96] (Fig. 5). Although several potential genes related to sheep tail morphology have been identified, the causal variant(s) and mutation(s) of these high-ranking candidate genes are still elusive and need further investigation. The *T-box transcription factor T* gene (also known as the *T* gene or *brachyury*) encodes a developmental transcription factor that was first discovered in mice [97]. It is involved in mesoderm formation and differentiation and regulates the number of caudal vertebrae and the tail length in various mammalian species, including sheep [98, 99]. The reason why the domestication process led to the selection of a phenotypic trait such as the long tail, which apparently lacks selective advantages, is still under investigation. A possible explanation could be the co-selection with other phenotypic traits, which are characteristic of domestic breeds, and are controlled by genes located on adjacent genomic regions [45].

In contrast to the result of the domestication process that favored long tails, such a trait is now considered a morphological feature of concern in breeding. This has prompted farmers to adopt drastic solutions such as docking the tails of newborn lambs. However, tail docking causes acute pain to lambs in their early stages of life and makes them more susceptible to parasitic infections, which in some cases leads to the death of the individual with consequent economic damage for farmers. Interestingly, several attempts have been made to reduce the tail size in fat-tailed and thin-long-tailed sheep breeds by using crossbreeding [83]. These innovative approaches could help to improve sheep breeding in the future and to lead to new and improved breeds.

### Presence of horns

Based on the presence of horns, sheep can be divided into three main groups: (1) both sexes carry horns but with those of the females being much smaller, as reported for wild sheep in Central Asia; (2) males have well-developed horns, females are polled, as in the case of most mouflons inhabiting the islands of Corsica and Sardinia; and (3) both sexes are polled, a condition typical of most domestic sheep breeds [100].

**Fig. 6:**
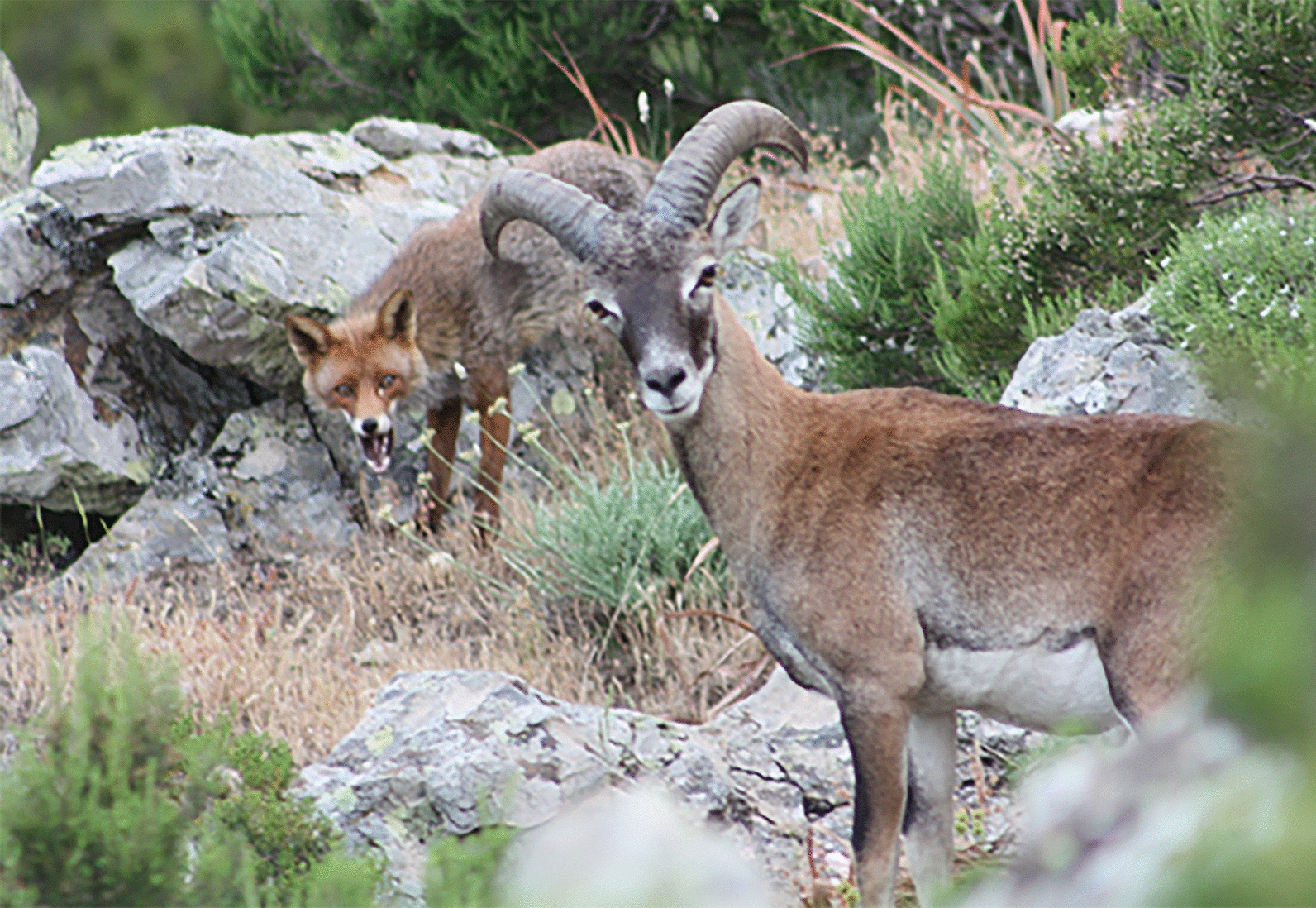
Young mouflon ram. The small growing horns are shown

**Fig. 7:**
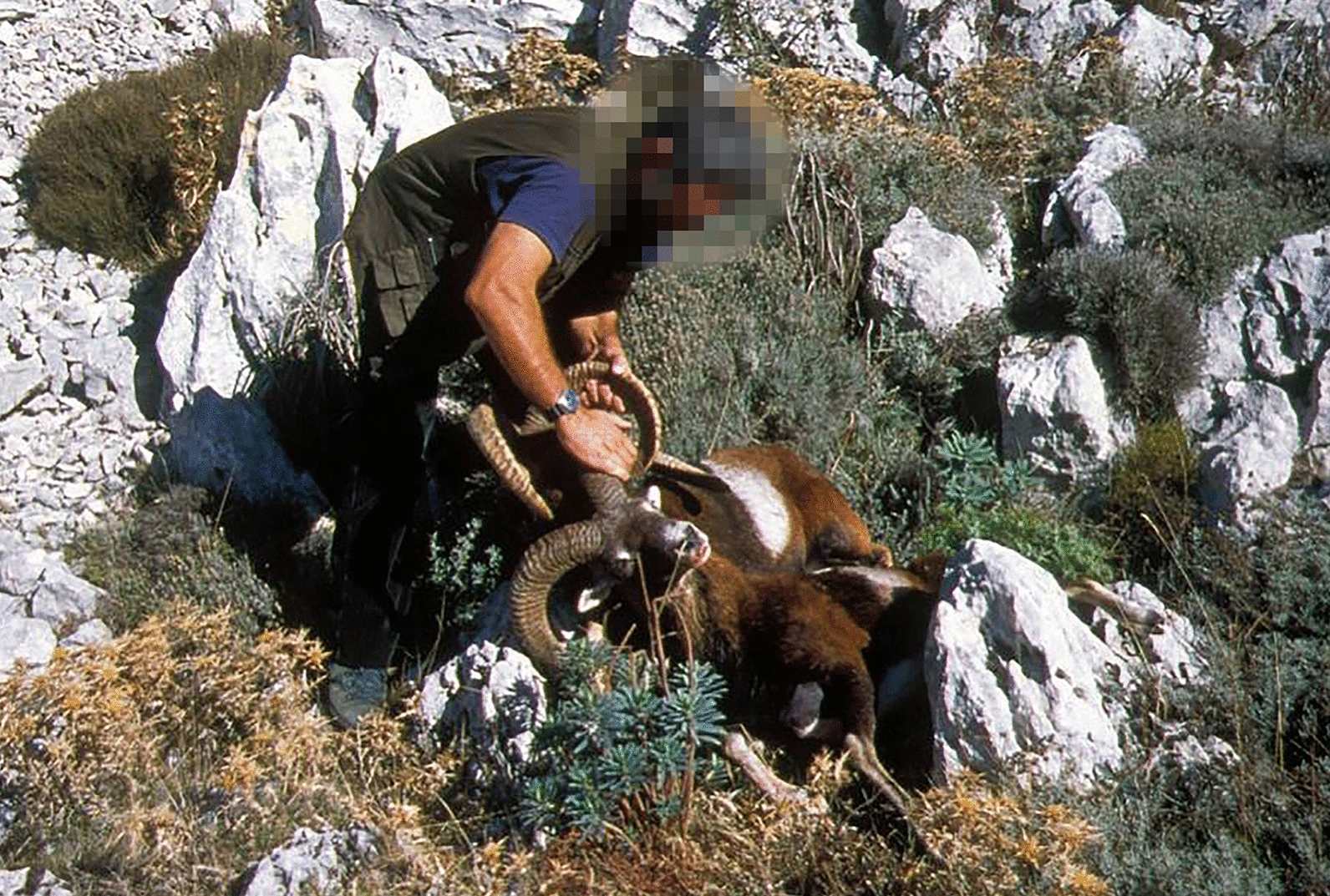
Mouflons with crossed horns after a fight. Human intervention was necessary to free the horns that got stuck during the clash

In the first year of life, horns grow linearly until puberty (Fig. 6). In the European mouflon, horn growth is influenced by the photoperiod, showing a circannual trend opposite to the plasma level of testosterone, with a maximum growth during the spring and summer and a significant reduction during the autumn and winter [101]. Horns, which represent the most characteristic trait of wild sheep, serve for intra-sexual competition and fighting. In this context, intra-sexual selection is an evolutionary process that shapes the phenotypic traits which are used in the competition between members of the same species (in general males) to gain opportunities to mate with the opposite sex. Therefore, horns are an important trait from an evolutionary perspective although they can be associated with reduced survival of single individuals [102, 103]. Indeed, horns confer benefits to males during fights for mating, but can be damaged or, even worse, become locked together (Fig. 7) leading to certain death of both males (personal observation).

Moreover, horns are an important trait also for animal breeding since they represent a potential danger to both humans and farm animals. In the event of a clash between individuals within the same farm, the presence of horns can cause serious bruises and wounds that reduce the quality of the meat. For this reason, many farmers prefer animals without horns and adopt the practice of dehorning males to reduce the risk of injury. In such a context, breeding for polledness represents an animal-friendly alternative to surgical dehorning [104, 105]. European legislation prohibits dehorning as a routine treatment, but local authorities can permit some exceptions. Hence, understanding the genetic background involved in the development of horns is crucial for the selection and improvement of sheep breeds.

In sheep, horn formation starts during the embryonic period. Over the years, several studies have identified a set of putative loci that are associated with the sheep horn status, although the underlying molecular mechanisms have not yet been elucidated. Particularly, the prominent role of the *RXFP2* gene in the presence/absence of horns in sheep has been suggested [67]. Recently, a correlation between the Wnt signaling pathway and horn formation in sheep has been proposed. Indeed, the horns of ruminants originate from the NCC and the Wnt signaling pathway is essential for regulating the fate, migration, and proliferation of cranial NCC [106]. Based on this evidence, we cannot exclude that some key genes and pathways involved in the development of horns could be attributable to the NCC hyphotesis.

### Intermediate phenotypes of the hybrids between domestic and wild sheep

For researchers interested in reconstructing the history of animal domestication from its earliest stages, identifying the phenotypic responses to domestication remains an important and long-standing issue. In this sense, hybrids between domestic and wild species represent a powerful model for investigating the phenotypic variations resulting from the domestication process and for evaluating the impact of human control on them. Indeed, they provide useful information on the mode of inheritance of phenotypic traits, since the phenotype of hybrids can be more similar to one parent than the other, intermediate or distinct from both of them.

The production of wild/domestic sheep fertile hybrids is an occasional event that can occur in geographical areas that host sheep farms and free ranging mouflons [107]. Such hybrids were already known during the Roman empire, when they were called *umbri* as reported by Pliny the Elder [108]. A necessary and conducive condition to crossbreeding is the overlapping of the reproductive period between domestic and wild sheep [109, 110]. Another cause of the production of hybrids is human-mediated crossing, with often deleterious and sometimes irreparable effects on the genetic integrity of natural populations. As reported by Uloth [111], after the arrival of European mouflons from Corsica and Sardinia in 1732 at the Zoo in Vienna, some mouflon populations were introduced in several European countries and deliberately crossed with primitive domestic breeds to improve robustness and trophy size. Because of these crossings, the presumed purity of most European mouflon populations has been debated for decades and is still highly questionable [6, 112, 113]. The interest in hybrids is motivated by the fact that hybridizations often favour an increase in the vigour of the single individual and in the development of the offspring, which are characteristics that are advantageous from a reproductive point of view [114]. Hybrid vigour appears as an expression of superior characters to those possessed by both parent stocks and manifests itself in faster growth, larger size, better productivity, superior vitality, better resistance to disease and in other ways [115].

**Fig. 8:**
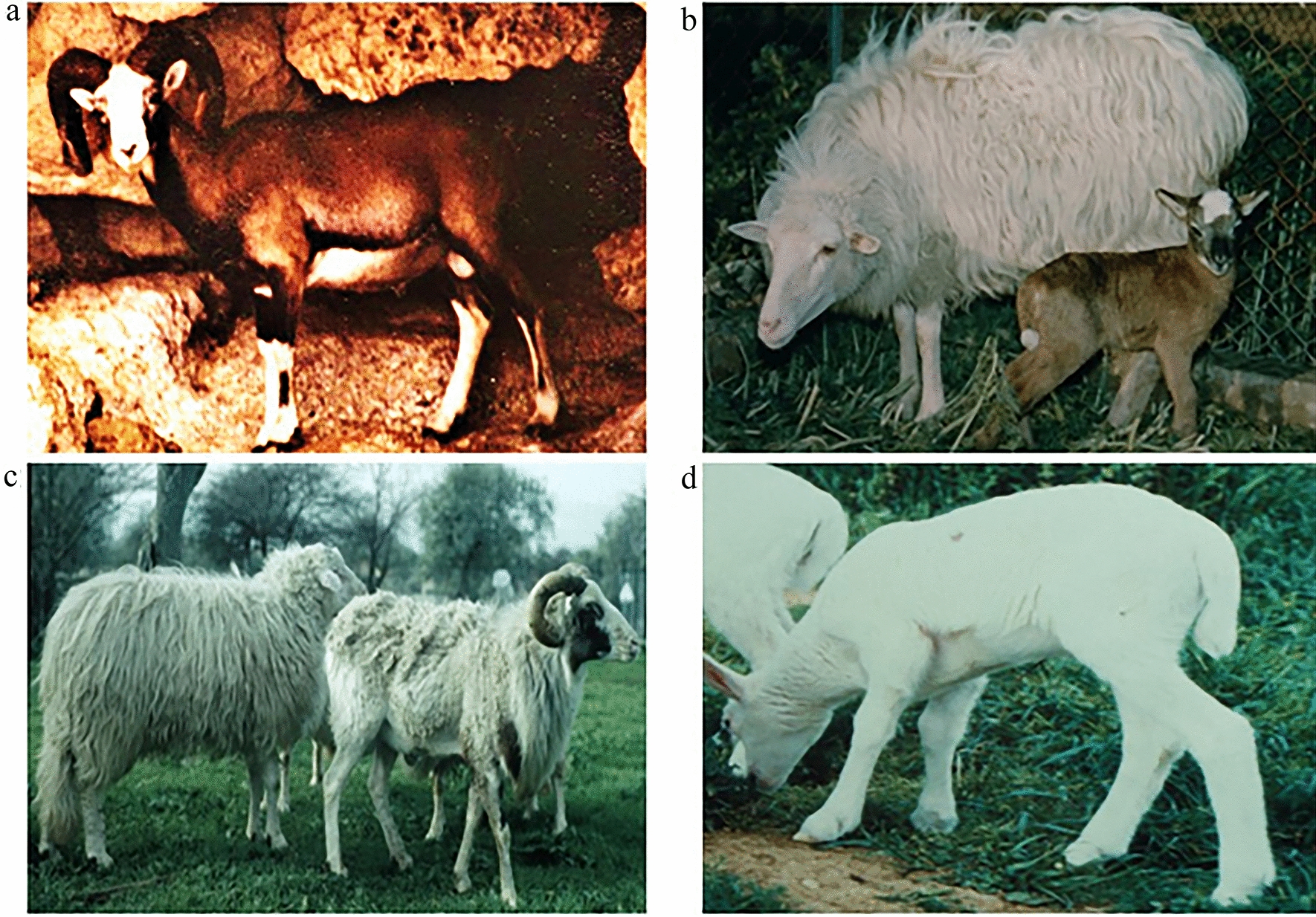
Intermediate phenotypes in mouflon x domestic sheep hybrids. Natural hybrid with white head and absence of sella (**a**); artificial hybrid with white head and tail (**b**); artificial hybrid with annual shedding (**c**); artificial hybrid with a tail of intermediate length (**d**)

The hybrid sheep × mouflon may have intermediate phenotypes for traits including coat color, shedding and wool, shape and size of the tail and the horn (Fig. 8). Studies on artificially produced hybrids have shown that coat color ranges from white with light brown spots to brown with small white areas, the tail has an intermediate length between that of the two parent species and the color of the horns ranges from white to brown [116]. These phenotypic changes become less and less evident with each generation until they become very difficult to detect. Such a trend can be explained by considering that when these polygenic traits escape from the strong control of human artificial selection (that reduces the number of alleles and increases the frequencies of homozygosis), the presence of wild allelic variants in the hybrids may lead to the manifestation of the codominant phenotypes which had disappeared during the domestication process. In introgressed populations, this phenomenon can appear less evident.

Although it can be a useful tool for a first-level discrimination, recognizing a hybrid individual based on phenotype only can be extremely difficult. In order to check the presence and the level of potential introgression of the domestic component into hybrids, molecular analyses represent a valid and accurate method of investigation. Molecular approaches that are able to discriminate between wild, domestic and hybrid individuals have been developed in the last decades [107, 112, 117–119]. Such analyses are based on the often combined use of mitochondrial and nuclear markers such as microsatellite and single nucleotide polymorphisms, which represent a rapid and effective tool and are very useful in preserving and managing wild ungulate populations. Indeed, they help to limit the spread of domestic genes that could dilute the ancestral genes that characterize the wild species.

## Conclusions

The phenotypic transition from a wild ancestor to a domestic descendant occurs through a long and complex process that is characterized in the early phase by the selection of tame individuals, more inclined to interact with humans. This first step leads to the genetic co-selection of a set of phenotypic traits which characterize most of the domesticated species. Then, a second human-mediated selection takes place to improve productivity. Similarly to selection practices in the past, the genetic traits of today’s domestic species are progressively and deliberately shaped according to human needs, but with increasing attention to animal welfare and dietary preferences of society. An example is represented by wool, which is almost completely replaced by cheaper and more versatile artificial textile fibres. The contribution of wool has declined by about 50% over the past 20 years and today represents about 1.5% of the total fiber production worldwide [120]. The costs of shearing sheep and producing wool have increased significantly, and have become unsustainable for breeders who are therefore turning their attention towards the meat and milk market. The renewed interest in primary products such as meat and milk at the expense of secondary ones (wool) represents a real reversal of the trend compared to the direction followed during the selection process for domestic characteristics. Furthermore, key points to consider are the growing interest in animal welfare, which opposes any practice that causes suffering to animals, such as tails and horn cutting, and the greater attention to a more balanced and low-fat diet that has reduced the demand for sheep with fat tails compared to the past. To limit the costs of shearing the coat, dehorning and tail cutting, it is not excluded that sheep farming may return in the future to the wild phenotype with natural shedding, short and thin tails and the absence of horns. In this journey back in animal husbandry, technological progress in the field of genomics can help to identify sheep breeds that carry the desired genetic characteristics that can be transmitted to future generations through reproduction.

## Data Availability

Not applicable.
